# Perceptions about controlled human infection model (CHIM) studies among members of ethics committees of Indian medical institutions: A qualitative exploration

**DOI:** 10.12688/wellcomeopenres.17968.2

**Published:** 2023-02-28

**Authors:** Abhishek Sharma, Aditi Apte, Medha Rajappa, Manjulika Vaz, Vina Vaswani, Shifalika Goenka, Samir Malhotra, Rashmi Sangoram, Subitha Lakshminarayanan, Suganya Jayaram, Jayanthi Mathaiyan, Khadeejath Farseena, Prarthna Mukerjee, Surinder Jaswal, Amol Dongre, Olinda Timms, Nusrat Shafiq, Rakesh Aggarwal, Manmeet Kaur, Sanjay Juvekar, Amrita Sekhar, Gagandeep Kang

**Affiliations:** 1 Postgraduate Institute of Medical Education and Research (PGIMER), Chandigarh, Chandigarh, 160014, India; 2 KEM Hospital Research Centre, Pune, Maharashtra, 411011, India; 3 Jawaharlal Institute of Postgraduate Medical Education and Research (JIPMER), Puducherry, Puducherry, 605006, India; 4 St John's Medical College, Bengaluru, Karnataka, 560034, India; 5 Yenepoya University, Mangalore, Karnataka, 575018, India; 6 Centre for Chronic Disease Control (CCDC), Delhi, Delhi, 110016, India; 7 Tata Institute of Social Sciences, Mumbai, Maharashtra, 400088, India; 8 Pramukhswami Medical College, Karamsad, Gujarat, 388325, India; 9 Translational Health Science and Technology Institute, Faridabad, Haryana, 101213, India; 10 Christian Medical College, Vellore, Tamil Nadu, 632004, India

**Keywords:** Risk benefits, ethics, viral strain, vaccine development, human challenge studies

## Abstract

**Introduction: **Controlled Human Infection Model (CHIM) studies provide a unique platform for studying the pathophysiology of infectious diseases and accelerated testing of vaccines and drugs in controlled settings. However, ethical issues shroud them as the disease-causing pathogen is intentionally inoculated into healthy consenting volunteers, and effective treatment may or may not be available. We explored the perceptions of the members of institutional ethics committees (IECs) in India about CHIM studies.

**Methods: **This qualitative exploratory study, conducted across seven sites in India, included 11 focused group discussions (FGD) and 31 in-depth interviews (IDI). A flexible approach was used with the aid of a topic guide. The data were thematically analyzed using grounded theory and an inductive approach. Emerging themes and sub-themes were analyzed, and major emergent themes were elucidated.

**Results: **Seventy-two IEC members participated in the study including 21 basic medical scientists, 29 clinicians, 9 lay people, 6 legal experts and 7 social scientists. Three major themes emerged from this analysis—apprehensions about conduct of CHIM studies in India, a perceived need for CHIM studies in India and risk mitigation measures needed to protect research participants and minimize the associated risks.

**Conclusion: **Development of a specific regulatory and ethical framework, training of research staff and ethics committee members, and ensuring specialized research infrastructure along with adequate community sensitization were considered essential before initiation of CHIM studies in India.

## Introduction

Controlled human infection model (CHIM) studies, also known as human challenge studies, involves a calculated and measured introduction of an infectious organism into consenting healthy adult study participants. These studies can help understand the pathophysiology of a particular infectious disease in response to different doses of an infectious agent, the development and nature of immunological responses in humans to a pathogen, and the response of infection to the vaccines and therapeutic agents ^1, 2^. As CHIM studies have less variability of disease than in natural infection, it allows rapid testing of vaccines and drugs, making the process of their development more efficient and cost-effective. In recent years, CHIM studies have facilitated the development of vaccines against several diseases, such as typhoid, yellow fever and cholera ^3^. In the ongoing pandemic of coronavirus disease 2019 (COVID-19), the use of CHIM studies for severe acute respiratory syndrome coronavirus-2 (SARS-CoV-2) infection had been proposed ^4^ and initial studies to establish the inoculum dose needed to produce a reproducible infection have begun in the United Kingdom ^5^. In low or middle-income countries (LMICs), despite high burden of infectious disease, CHIM studies have been carried out infrequently ^6, 7^, in Kenya ^8^, Malawi ^9, 10^ and Thailand ^11^ for diseases such as malaria, shigella, and streptococcal pneumonia.

Conduct of CHIM studies raises important ethical issues. A disease-causing pathogen for the purpose of the study is intentionally inoculated into healthy, consenting adult volunteers, with no individual benefit to them ^1^ a possible violation of the principle of non-maleficence. The degree of it could vary depending on the pathogenicity of the virulent introduced, the dosage and the preparedness to manage its onslaught on the body and the effectiveness of the treatment available This issue of non-maleficence is heightened further when there is no treatment available for the infection. Is it justifiable to infect a volunteer and place them at risk for the benefit of humankind even with their consent? In the LMIC setting, there is an additional risk of exploitation of study participants due to their lower educational status and extreme poverty ^12^. These are ethically contentious issues which require further exploration. Hence, CHIM studies, particularly for COVID-19, have been a subject of much debate and disagreement in scientific circles as well as the lay community ^13, 14^. To address these issues, the World Health Organization (WHO) set up a Working Group to assess the ethical acceptability of CHIM studies in COVID-19 vaccine development ^15^ and has also published a guidance on ethical conduct of CHIM studies ^16^.

Although CHIM studies have not commenced in India, several deliberations on the ethical aspects of such studies, involving biomedical researchers, clinicians, policy makers, regulators, and ethics and legal experts from the country, as well as international experts with previous experience of doing such studies, have been held since 2017. There have also been several publications on various aspects of CHIM studies by Indian researchers ^17– 19^. More recently, Indian Council of Medical Research convened a meeting on the issue of CHIM studies in the context of the ongoing COVID-19 pandemic with several international experts ^20^. These consultations have led to the realization that it is important to understand the perspectives of various stakeholders in CHIM studies. While a previous study has captured the perspective of the community on CHIM studies ^18^, the need to understand the perspectives of members of institutional ethics committees (IECs), whose main objective is to protect the ethical rights of the research participants, was considered important. The present study explored the views of members of ethics committees about CHIM studies in their context. It was carried out amongst consenting members of IECs from multiple health care research institutes in India with experience in conducting clinical trials

## Methods

### Study sites and investigators

As indicated above, the participating sites were selected from among institutions with experience in conducting clinical trials and whose IECs are registered with the Drug Controller General of India and the Department of Health Research, Ministry of Health and Family Welfare. The study sites included both government and private-owned institutions: KEM Hospital Research Centre (KEMHRC), Pune (site 1); St. John Research Institute (SJRI), Bengaluru (site 2); Yenepoya University (YU-deemed to be University), Mangalore (site 3); Centre for Chronic Disease Control (CCDC), Delhi (site 4); Jawaharlal Institute of Postgraduate Medical Education and Research (JIPMER), Puducherry (site 5); Postgraduate Institute of Medical Education and Research (PGIMER), Chandigarh (site 6). All the site investigators had a doctoral or postgraduate degree in a biomedical field, and experience of conducting qualitative research and serving on an ethics committee. They were also familiar with the IEC members at their sites through their prior interactions. The investigators included a mix of both genders.

### Study design

**Figure 1.**
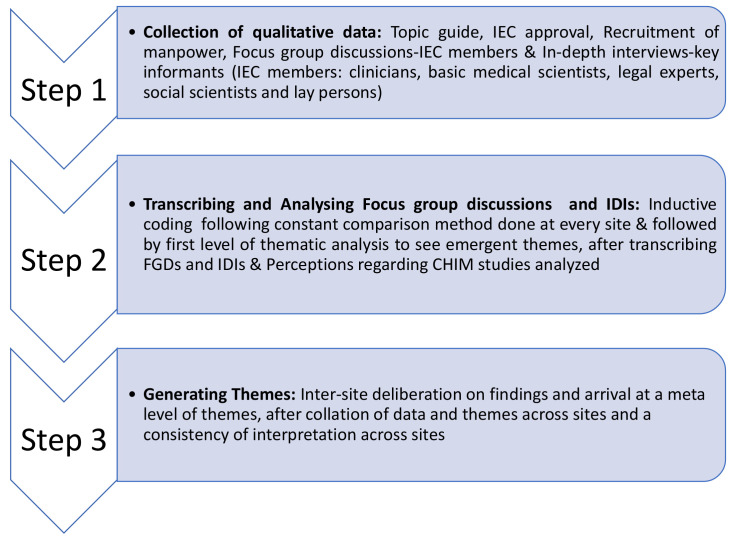
Summary of methodology.

**Figure 2.**
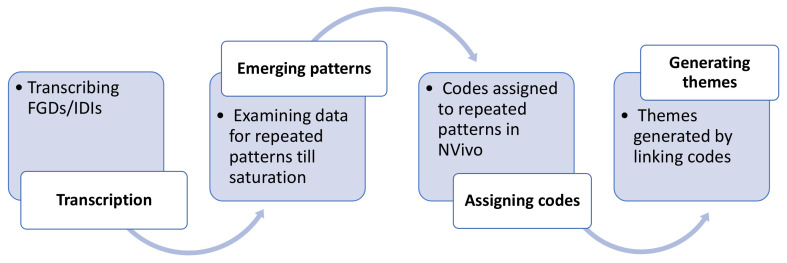
Generating themes.

This was a multi-centric qualitative exploratory research study, conducted between February 2021 to July 2021 involving IEC members of six institutions. The present study employed a grounded theory method and an inductive approach which involved ^21^ gathering data through focus group discussions (FGD) and in-depth interviews (IDI). This exploration is referred to as 'grounded' since they are based on the participants' own explanations or interpretations. An inductive approach ensures that the researcher begins with an almost blank slate and considers the opinions of the participants rather than imposing one’s own. This was important as CHIM studies are new and de novo rather than a priori ethical responses to them were being sought. Figure 1 and Figure 2 summarizes the methodology of this study.

### Participants

At each site, all the IEC members were invited to participate in the study—either in FGD and/or IDI. The study participants belonged to diverse fields, covering the range of expertise of mandated IEC members—clinicians (CN), biomedical scientists (BM), social scientists (SS), legal experts (LE) and lay persons (LP), with rich experience and expertise in their respective roles. Site-wise details of participants are provided in Table 1. IEC members who were not able to participate cited reasons such as lack of time due to increased institutional responsibilities during the COVID-19 pandemic, poor health and COVID-19 illness.

**Table 1.** Site-wise details for conduct of the study.

Site ID	Number of individuals approached		Number of individuals who participated		Number of FGDs	Average time for FGD in minutes	Number of IDIs	Average time for IDI in minutes	Mode of interaction
	FGD	IDI	FGD	IDI					
Site 1	11	9	10	9	2	60	9	45	Virtual or in- person
Site 2	15	6	9	3	1	95	3	60	Virtual
Site 3	12	9	9	6	2	84	6	40	Virtual or in- person
Site 4	8	0	6	0	1	50	0	0	Virtual
Site 5	28	14	18	13	2	90	13	45	Virtual or in- person
Site 6	21	0	15	0	3	58	0	0	Virtual

### Approvals, ethics and consent

The study protocol and tools were approved by the respective IECs [KEMHRC: KEMHRC/RVM/EC/2061 dated 29 Jan 2021; SJRI: SJMC IEC 19/2021 dated 7 Jan 2021; YU: YEC-1/53/2021 dated 13 March 2021; CCDC: CCDC_IEC_04_2021 dated 26 Feb 2021; JIPMER: JIP/IEC/2021/09 dated 19 Feb 2021; PGIMER: PGI/IEC/2021/000442 dated 26 March 2021]. A written informed consent was obtained from all IEC members who agreed to participate in either FGD or IDI, or both. Participation was voluntary and not remunerated. The study was conducted in accordance with the National Ethical Guidelines for Biomedical and Health Research involving Human Participants, 2017 ^22^. 

### Data collection

The data were collected using FGDs that mirrored the deliberative process of decision-making in IEC meetings, and semi-structured IDIs to get more in-depth understanding of perceptions of individual IEC members. In all, 11 FGDs and 31 IDIs were conducted across all the sites. The site-wise details regarding the FGDs and IDIs are provided in Table 2. Of the 95 individuals approached for the FGDs, 67 (71%) accepted to participate. For the IDIs, 38 individuals were invited, and 31 (82%) accepted to participate. The FGDs lasted for 40–90 minutes and the IDIs lasted for about 40–60 minutes. Two topic guides, one each for FGDs and IDIs, were developed, after discussion with the site investigators and pilot testing at one site (available as extended data ^23^). Each site was permitted to modify the guides and to add site-specific probing questions based on the local context. Due to the ongoing COVID-19 pandemic, some of the FGDs and IDIs were conducted either online, or in person while following COVID-19-appropriate protocols. All the study tools were in English, since this was the language of communication of all the IECs. The FGDs and IDI were moderated by the respective site investigators. Each interviewer was supported by an associate, who was also experienced in qualitative research methods and took notes during the interviews or discussions. The FGDs and IDIs were audio-recorded with permission of the study participants; the audio recordings were transcribed verbatim in English and transcriptions were rechecked.

**Table 2.** Centre-wise age and gender distribution.

Institute	Age range in years	Men:women ratio
Site 1	35–79	3:8
Site 2	38–66	5:6
Site 3	35–79	7:2
Site 4	40–75	3:3
Site 5	35–69	8:12
Site 6	30–69	8:7

Before each FGD, the participating IEC members were provided uniform information (vetted by experts) on CHIM studies for prior reading (available as extended data ^24^) and a brief specifically-designed PowerPoint presentation that included background information on CHIM studies and examples of such studies conducted elsewhere.

During the FGD as well as IDIs, the enquiry focused on possible challenges in the conduct of CHIM studies in India—for IECs as well as the investigators, eligibility criteria for unbiased selection of study participants, need for modifications in the ethics committee review process for CHIM studies, need for training, compensation or reimbursement for participation, involvement of social media, community engagement activities and need for CHIM studies for COVID-19. Additionally, demographic details and information about qualification, years of experience as an ethics committee member were also collected. The IEC members were assured that there were no right or wrong answers to the questions, and that the FGDs and IDIs were being done solely to understand views and perceptions on the ethical aspects of doing CHIM studies in India. FGDs and IDI played a complementing role. While FGDs provided the dynamic process of discussion and deliberation similar to the real world functioning of IECs in reviewing research proposals, the IDIs helped to provide a more in-depth perspective of individual members. Provision of allowing IDI also allowed views of a member who could not make it on the day of the FGD to express his/her views separately.

The details of study methods are depicted in Figure 1.

### Data analysis

The data at each site were subjected to thematic analysis using grounded theory and an inductive approach ^25^. FGDs and IDIs were conducted till data saturation or redundancy criteria were reached. The transcripts were rechecked and verified by researchers within the site team. These were de-identified and then shared with the core-team. Transcripts were critically assessed to identify and construct the emergent themes. Coding was an iterative process done at individual sites (either manually or using a qualitative analysis software [NVivo 12]) and across sites using the methods of constant comparison to arrive at mutually-agreed, comprehensive codes without duplication. As IDIs were meant to explore the issues discussed in the FGDs, data from both FGDs and IDIs were analyzed together, and were not compared with each other. Thereafter, in a workshop conducted across sites, presentations were made where codes were discussed and de novo codes arrived at to establish patterns or relationships in the data. Emerging themes from sub-themes were deliberated upon, differences resolved, and consensus arrived at. The process of generating themes is depicted in Figure 2. The quotes were labelled with the role of the IEC member and the site number. *e.g.* BM01 indicates basic medical scientist from site 1.

## Results

### Demographic characteristics of IEC members

A total of 72 IEC members participated in the study that included 34 men and 38 women members with an age range of 30–79 years [ Table 2]. The participants included 29 CN, 21 BM, 7 SS, 6 LE and 9 LP. The characteristics of the participating IEC members is shown in Table 3.

**Table 3.** Characteristics of the members of the institutional ethics committees (IECs) who participated in the study.

Years of experience as member of IEC	Role in IEC					Total number
	BM	CN	LP	LE	SS	
1-5	8	11	5	1	2	27
>5 to 10	6	7	2	1	1	17
> 10	7	11	2	4	4	28

*BM- basic medical scientist, CN- clinician, LP- lay person, LE- legal expert, SS- social scientist*

### Findings

Across all sites, IEC members were extremely articulate and freely expressed their views and counterpoints during the FGDs and IDIs. There was active listening and respectful interjections, openness to each other’s perspectives, and a willingness to reconsider early positions and move to a practical problem-solving mode. Members engaged actively with the subject and there was always more than one hand up in an FGD, asking to speak. They took notes to help keep track of the discussion points, and each person shared multiple points on each question raised. This shows that group members knew each other well and possibly follow similar processes in their working as an IEC. The topic, though unfamiliar to most, was refreshed with every probing question. Perceptions were insightful and well considered.

The thought processes expressed had three major emergent themes. The first theme was “Apprehensions towards the conduct of CHIM studies in India”, given that CHIM studies involve complexity, expertise, and possible ethical challenges and risks particularly in the social and economic context of India, with a scope for misuse and volunteer coercion. The second theme was “Perceived need for CHIM studies in India” with apparent scientific and public health benefits. This was especially in the context of an urgent need for a vaccine in a health emergency such as COVID-19. The third theme, “Proposed measures for risk mitigation in CHIM studies”, covered methods, structures, processes and regulations which would prevent untoward incidents, and minimize harm and risk to the study participants.

### Theme 1 – Apprehensions towards conduct of CHIM studies in India

The participating IEC members across sites, hereafter referred to as ‘members’, shared that CHIM studies were to be considered as ‘highly specialized’ studies, requiring specialized dedicated facilities, competent and qualified research teams and medical professionals. In CHIM studies, infection is deliberately introduced, and participants may not fully comprehend the risks associated with this. Given that CHIM studies were new to healthcare professionals as well as IECs, the members expressed uncertainty about the lay public being sufficiently aware, much less comprehending CHIM studies.

A particular concern expressed with CHIM studies was the risk of infection causing possible long-term consequences which may not be fully known, *e.g.* the risk of long-COVID following CHIM studies on COVID-19 ^26^, necessitating stringent monitoring and long-term follow-up. There was also apprehension regarding IEC members’ added responsibility since the diseases being studied may not have a treatment and may pose a serious threat to the study participants. This was of greater concern to the legal experts serving on IECs. The scope for misuse and volunteer exploitation was considered high for the following reasons: CHIM studies could attract lucrative funding, which could induce medical professionals and institutions without adequate expertise and structures for oversight and volunteer safeguards, to undertake these studies. Members continuously reiterated the context of India’s socio-economic deprivation, where such studies could lead to coercion of vulnerable individuals to participate. Impoverished men and women may enroll to tide over extreme poverty and provide for their families.

Institutions and investigators conducting CHIM studies need special expertise, advanced medical care facilities and quality multidisciplinary specialists. There is a high chance of institutions and professionals without the required and requisite expertise and facilities jumping in to do CHIM studies. The other apprehensions expressed included risk of infection to the study team and the community at large, possible stigmatization of participants, lack of support from the general public and concerns of human right activists. Standardisation of the infectious strain used was a further concern.

Table 4 provides details of apprehensions expressed by the members, in the form of sub-themes with supporting illustrative quotes.

**Table 4.** Apprehensions of IEC members towards conduct of CHIM studies in India.

Subthemes	Specific concerns	Illustrative quotes
Lack of specialised research infrastructure and trained scientific workforce required for CHIM studies	- Stringent biosafety measures required for CHIM studies - Lack of formal training amongst research community - No prior experience with CHIM studies - Lack of adequate BSL3 safety labs and specialized Phase I clinical trial units	*“But I hope it is very seriously taken care of all the infrastructure* *has to be, you know, watertight, that's what I feel’’*– *CN01.* *“...the quality has to come to certain minimum global standards* *before we can allow it to happen now”* – *CN03*
Risk of exploitation	- Possible inducement of vulnerable study participants - Difficulty in understanding the risks and the benefits of study participation	‘ *’When you are selecting participants, it should be from a very* *diverse population. So that, the process of selection can be fair* *enough’’*– *LP01*
Safety of the study participants	- Risk of serious illness - Lack of rescue treatment - Lack of clear rules and regulations - Long-term safety issues	*‘’This has a very high stake, the normal person getting infected.* *And there is no way that you can afford a side effect. You have to* *pre-empt it and you have to treat it before it happens’’*– *SS01* *“Some of them who are lucky are escaping but others will go* *straight to the ICU because of the disease, of course in CHIMS,* *the strains are going to be very diluted. So, they may not have* *such bad, but one never knows. So, I feel that the risks are more* *than the benefits’’*– *CN01*
Challenge- Standardisation of viral strain `	- One needs to ensure that the viral strain does not cause severe disease - Difficulty in standardizing viral strain in the instance of SARS-CoV-2	*“We're talking only in terms of strains alone for those strains* *of virus, which are incapable of causing, I mean, in which the* *replication of the virus does not occur once inside the body.* *So, I'm alright with a, you know, like adenovirus strain, or other* *baculovirus strain, and I'm okay with mRNA virus strain”* – *BM01*
Risk of infection to the study team	- Risk of infection to the study staff -Lack of standard operating procedures in medical research institutions - Lack of trained study staff	*“It can actually spread to the doctors also when you are doing* *the research. So he needs to be covered (insured) for doing* *these things…”* – *CN03* *“So, the challenge faced by Investigators...they are doing it at* *great risk, …..”* – *CN05*
Lack of specific regulatory framework	- Current rules are inadequate for CHIM studies - Need for stringent monitoring of CHIM studies - Modified AE/SAE compensation - Third party insurance	*“We have never thought about exposing people, healthy* *volunteers for such a long time, So there's no law as such in* *place for today. Probably they will have to make some laws or* *have to tweak some laws, they'll have to make specific provisions for this”* – *LE01*
Unfamiliarity about CHIM studies amongst the IEC members	- Lack of training and research integrity - Additional responsibility - Lack of prior experience	*“Forget about the public or the lay person even, I am sure* *the medical fraternity itself is not aware of what a CHIMs is. I* *must mention that I wasn’t very aware about what the CHIMs* *are, and I am beginning to understand it slowly as the FGD is* *progressing, being a basic medical scientist.”* – *BM02* *“EC’s have to be strengthened to review more CHIM studies…”* *–CN03* *“Like I can say like it will be a more burden for ethics* *committee….. Because continuous review is required .”* – *CN03*
Possible mistrust and lack of support for CHIM studies from the general public	- Lack of awareness regarding CHIM studies - Public mistrust in healthcare - Wrong information in social media. - Prior experiences with medical failures - Opposition by human right activists	*“We will need some time to prepare the society”* – *CN05* *“And then of course, there are the societal risks in that if you* *do such kind of study and it fails, then there's a total erosion of* *trust in the study’’* – *LE01* *“I really am thinking about the human rights activists and the* *environmentalist. Because there's a major crop of these people* *in India these days, and they resort to your information in social* *media of spread of information in social media. And it may be* *one hell of a thing to deal with. very seriously anything. ’’* – *LE01*
Spread of infection to the community	- Possibility of infection spreading to other members of the community - Volunteers and researchers may face stigma and discrimination during or after the study	*“...If I am going to infect a healthy person with CHIM...with* *COVID 19...again he would be quarantined and the family will* *be quarantined...the community will say..go..don’t come near us.* *That is going to add to the stigma and discrimination”* – *CN03* *“thing which is not yet discussed is this whole issue of stigma. * *…when you look at covid or take HIV infection, those who get* *infected do not want to reveal that they are infected …[therefore]* *getting infected with a disease artificially for research- sake is a* *big challenge for me”* – *BM02*

### Theme 2 – Perceived need for CHIM studies in India

Despite the apprehensions, given that animal models are either not available or not reliably applicable to all human pathogens, members felt that it is necessary to do CHIM studies in India, so that India does not get left behind in science, especially considering our country’s special needs and pathogens.

*“CHIM studies are justified when good animal models for a given infectious disease are not available and is a more authentic model for studying pathophysiology of an infection” –BM01*

The members felt that if the risks to an individual could be adequately counterbalanced and justified by benefits to the community, the overall benefit-risk ratio of CHIM studies could be improved. They quoted the history of vaccine development and how research which might have been risky had led to millions of lives being saved. The current situation of COVID-19 and the race to find a reliable, effective vaccine was continuously discussed and debated. Members felt that CHIM studies could help identify early leads in the course of development of COVID-19 vaccine and given the current circumstances, it would be easier to recruit volunteers for CHIM studies since people recognize the need for urgent vaccine development.

“ *It is also proved through the years, as you're rightly said, right, since the days of smallpox, but it is a fast tracking of getting the vaccine a little earlier, especially in this type of pandemic that we are going through. And we need quick results because the mortality has been too high’’ –CN01*
“ *COVID pandemic is a very good example for CHIM-like studies. We could come up with [an]immediate good vaccine with these 4-5 vaccine [candidates]*” *–CN06*

Some members persisted with the view that CHIM studies should be conducted only for such infections where rescue treatment is available, or where the infection is self-limiting. Some BM members felt that infection with agents for which no treatment is available poses a major risk to the volunteer in the event of serious illness, and they suggested that CHIM studies should be conducted only if absolute participant safety can be assured.

Members grappled with the question of whether CHIM studies should be conducted for diseases of public health priority, *i.e.* those that affect a large number of people, such as in the case of pandemics. SS and LP members, in particular, believed that CHIM studies should be conducted only in emergency situations such as the current pandemic.

*...“not always but only during such times (public health burden)*” – *SS03*
“ *In the emergency situation, it is very much needed” –LP03*

As for the role of CHIM studies in the Indian context, members opined that despite a high burden of infectious diseases in India, CHIM studies have not been conducted in India, leading to our dependence on research conducted elsewhere for vaccine and drug development.

### Theme 3 – Proposed strategies for risk mitigation in CHIM studies

The members felt that appropriate structures, and regulations required to protect study participants and to prevent abuse and misuse of CHIM studies are presently not available in India. Such guidelines are urgently needed to set standards for eligibility of institutions and investigators for CHIM studies.

Additionally, stricter systems of participant selection; stringent consent procedures involving counselling and ensuring comprehension by not only the participant but also their family members; reduction of risk of infection of others, e.g. family members and/or workers in the research facility by ensuring proper infection control and safety measures in the facility through strict biosafety and follow-up systems; and, specific insurance safeguards and specific compensation structures are needed.

It was suggested that this could be done by a central body (such as the Indian Council of Medical Research) bringing in strict regulations on the conditions and requirements of institutions who could do these studies, the participant protection needed and the responsibilities of IECs. Several members insisted that conduct of such studies could be restricted to only those institutions that have the required facilities and expertise, and experienced IECs.

The members further added that for CHIM studies on viral disease, because of absence of drug treatment and the wide spectrum of clinical consequences, a separate and tighter regulatory framework is required. Details of the sub-themes under required safeguards and suggestions are as follows:

**Specialized infrastructure**: Members stressed the need for physical, laboratory and waste management facilities appropriate for the biosafety level of the pathogen involved, and infrastructure and protocols for post-exposure prophylaxis. It was considered important to establish a standalone set-up with 24 × 7 access to healthcare, and living facilities.

*“So, it [institution] should have a facility, where research is given a priority, and also have all the facilities for treatment of any such complications and [professional] expertise who can understand the infectious disease in a better way” –BM05*

CN and BM, in particular, suggested that CHIM studies should be conducted in Phase 1 clinical trial units with provision for monitoring, isolation and recourse to advanced medical care as and when needed.

**Specific regulatory guidelines:** The members, especially BM and LE, commented that current regulations for clinical trials may not be adequate for conduct of CHIM studies considering the high risk involved in participation and would need significant modification. The members expressed the need for having stricter regulations for CHIM studies with provisions for monitoring in different phases of CHIM study, including participant recruitment, consent, data safety and post-trial phases. One LE expressed the opinion that laws may need to evolve with the advent of CHIM studies. Need for government regulations with regards to infrastructure requirements was expressed.CN members, among others, suggested the need for accreditation of IECs reviewing CHIM proposals and accreditation of centers carrying out CHIM studies.

*“..regulatory processes should be stricter in case of CHIM studies so that they are not misused” –CN03*
“ *We have never thought about exposing people, healthy volunteers for such a long time,..there's no law as such in place today. Probably they will have to make some laws or tweak some laws, [and] make specific provisions for this” –LE01*

**Training of researchers and IEC members:** The members emphasized the need for training in both the scientific and ethical aspects of CHIM studies. Interestingly, both CN and LP expressed that training of the research staff and IEC members about CHIM research is essential as India has no experience of conducting such studies. This would be a prerequisite for IECs in case they have to review proposals on CHIM studies. EC members were of the view that CHIM study proposals would require meticulous review and monitoring and involvement of a subject expert. It was also suggested that researchers/scientists/ethicists from around the world having prior experience in CHIM studies could be involved in training research staff and IEC members. Formal training with the help of a global body such as WHO was considered an option.

*‘’There has to be a separate training for the CHIMS. None of us have been trained, we have to have specific training by people who have done CHIM studies, I would invite the British to train us.’’ –CN02*

**Fair participant selection:** Considering the critical risk benefit ratio for CHIM studies, the members suggested the need for rigorous screening during selection and enrolment of study participants. It was considered a fair and ethical requirement for volunteers to be healthy, young, well-educated individuals able to understand the risks associated with the study.

*“People will have really need to be educated, educated to such a degree that people even the poor people, but who are you know, well educated, may participate’’–LE01*

**Informed consent:** The predominant view was that the informed consent process should be simpler but more interactive and rigorous than the current procedure and audio-visual recording should be done to ensure adequate comprehension and documentation. The patient information sheet should provide a thorough explanation about the nature and purpose of the research. There was a suggestion to include counselling by a psychologist to ensure that the participant comprehends the information and that there is no social pressure. Giving participants the opportunity and even encouragement to discuss their participation with their family members and /or family physician prior to consent was suggested. It was also proposed that a third party be involved in the process of obtaining consent to make it more transparent. Joint counselling sessions with volunteers and their families regarding the procedures, risks and follow up requirement was needed to ensure that consent is well informed and well understood.

*‘’I believe that the consent should be more in depth [and] in length. Even the language of the consent form should be such that an 8 ^th^ grade person should understand the essence of it.’’ –LE01*
*“Audio visual consent is mandatory. It should be made mandatory and a member who is external to the study should be a part … as the witness” –SS05*

**Compensation and study reimbursement:** Members felt that reimbursement and compensation to the participants of CHIM studies could prove to be a double-edged sword. While it could serve as fair financial compensation for the risks and inconvenience to the volunteers and their families, it could, on the other hand, lure and exploit poor people. Monetary compensation ran the risk of inducing volunteers from financially weaker sections to enroll in CHIM studies, particularly in India. To address this issue, the members suggested reimbursement only for loss of wages and travel rather than providing monetary compensation. Recruitment of volunteers from higher socio-economic strata was recommended to avoid financial inducement. However, this was a contested view, with some members endorsing that the participants in CHIM studies be provided extra benefits as the risks involved in such studies were considerably high.

*“I think extra benefits should be given because the amount of risk involved and the amount of psychological stress they may have during the study period, may be 10-20% more than whatever already involved to be approved by the Ethics committee to make sure that there is no inducement” –BM05*

These members also felt that the reimbursement and compensation amount in CHIM studies could be higher than in regulatory clinical trials and suggested that the authorities devise a new formula for compensation of CHIM participants. It would need to address all events that might occur during and after the study.

**Insurance coverage and indemnification:** As CHIM studies run the risk of spreading infection to the study team and the community, the members suggested that there should be a comprehensive insurance policy that covers third parties, such as the family members. They were however skeptical about insurance companies being willing to provide comprehensive insurance coverage and felt that pharmaceutical companies or the study sponsors may have to provide the necessary coverage. An additional point raised was regarding risks beyond the immediate third parties *e.g.* risk to the unborn fetus in case of a Zika virus CHIM, might have to be taken into consideration.

*“See the Insurance in India will not cover for the next generation...the insurance companies have to come up with some plans for CHIM...in this ability, I don’t think the insurance companies are going to cover them (family/third party affected by the study)” –LP03*

The members suggested that the entire study team as well as the IEC members involved in the review process should be indemnified considering the risk involved.

*‘’Investigator has to have a stronger indemnification because he is at the helm of everything.’’ –LE01*

**Stakeholder engagement and community dissemination:** The members felt that engagement activities were required to sensitize various stakeholders, such as the government partners, health care personnel and the general public. Methods suggested included educational campaigns such as public meetings, advertisements, discussions, and social media. Two-way communication with the community members was preferred, to provide clarity and build the community’s trust in research teams. Once trust is established, dealing with misinformation and erroneous perceptions about CHIM studies would possibly be easier.

*“Build trust among the community members. There is a common perception that such kinds of studies may have some hidden motive and common people are being used to achieve that motive” –SS06*

The members felt that collaboration with government bodies could increase the credibility of CHIM studies from the public perspective. The use of social media was proposed to disseminate positive information and to engage with misinformation about CHIM studies. However, they shared experiences about how false or negative information could get disseminated through social media networks and suggested responsible involvement of social media by the researchers right from the inception of the study to avoid this problem.

**Empowerment of ethics committees:** Considering the perceived high risk involved in CHIM studies, the members were clear that such studies would need very close monitoring by the IEC. IECs should be empowered with close oversight of the investigator team. Specific suggestions included frequent monitoring, real-time monitoring of participant recruitment by an IEC subcommittee, and monitoring beyond study completion for any (un)anticipated adverse events. Augmented authority or power of IECs in terms of decision making in suggesting substantial changes in the study design, recruitment and other procedures, site inspections, site closures, rigidity of competence of research teams, and long term follow up of facility, volunteers and family communities was suggested, to ensure the well-being of stakeholders. It was emphasized that the contribution of every IEC member was critical for decision making.

*“The monitoring will have to continue beyond the end of study dates here for whatever period, so all kinds of auditing need to be taken care of and periodic review has to be submitted if there is any other committee it has to be informed too.’’ –LE01*

**Figure 3.**
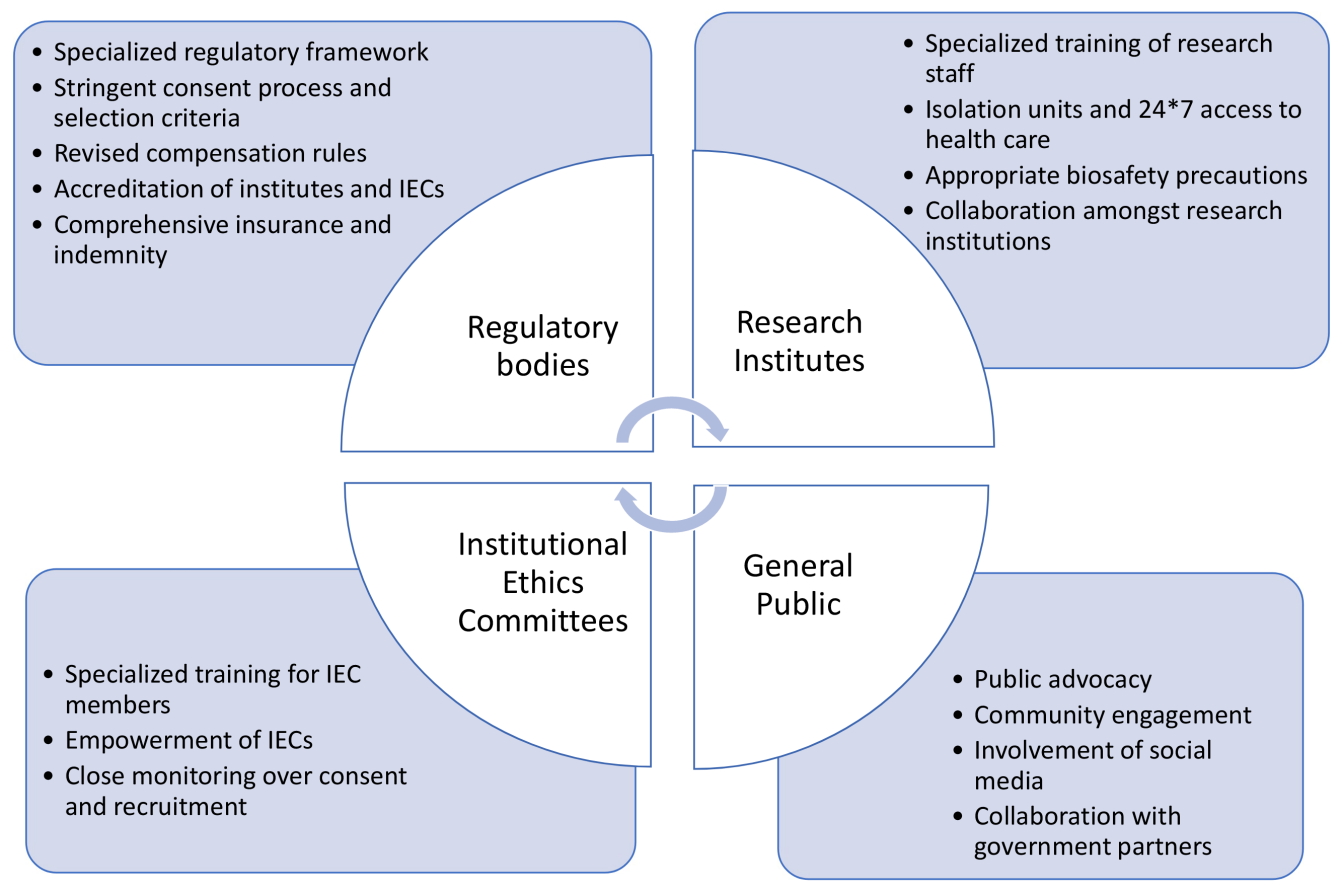
Proposed risk mitigation measures for CHIM studies.

A summary of the proposed risk mitigation measures from the perspective of regulatory bodies, research institutes, institutional ethics committees and general population is depicted in Figure 3.

Finally, the readiness of the country for CHIM studies was captured metaphorically as being a “ *Lamborghini on Indian roads*” indicating that the advanced level of expertise needed for the conduct of CHIM studies is much higher than that of existing clinical research in India.

## Discussion

Vaccines are important tools in the armamentarium of public health systems and with the advent of COVID-19 pandemic, the world has witnessed a desperate need for fast tracking vaccine development. CHIM studies provide a disease model for testing of vaccine candidates during early stages of clinical development and thus reduce the time to pivotal clinical studies ^27^. However, CHIM studies raise difficult ethical questions, in particular whether it is justified and permissible to infect a healthy volunteer with an infectious agent ^28^. Considering the burden of infectious diseases in India, introduction of CHIM studies may be needed in future and IEC members will be important stakeholders in ensuring the ethical conduct of CHIM studies and protecting the rights and wellbeing of study participants.

The results of our multicentric study indicate that IEC members from Indian healthcare and research institutions have several apprehensions regarding CHIM studies, such as vulnerability of our population due to poor socioeconomic status, lack of specialized research infrastructure, lack of specific training amongst researchers and IEC members, lack of stringent regulatory framework and public mistrust. The apprehensions extended to the possible risk of transmission of the experimental pathogen to family members of study participants and the wider community, as also the risk of progression towards severe disease; these were concerns of CN and BM members of the IECs, reflecting their real-life experience. A critical current concern in India is the breach of trust between the medical community and the general public, especially if there was any study-related death. Some of these concerns, such as the risk of infection and likelihood of exploitation of study participants, were also identified in a previous study conducted by Vaz *et al.* amongst lay public and key opinion leaders ^17^. These concerns are realistic considering ethical violations in the history of human clinical trials ^29^ and the fact that a clinician has an ethical commitment to benefit and protect the participant/patient ^30^. CHIM studies are different from first in human studies as these studies don’t just involve exposure to new drug but include establishment of a disease model in healthy humans using a standardized pathogen and then evaluating the effects of drugs and vaccines within the established disease model. This is in the context where people outside the study have already suffered from the pathogen as against a new drug which has never been used before. Overall, in the present scenario, implementation and acceptance of CHIM studies is challenging in an LMIC such as India, given the limited understanding of biomedical research and high vulnerability amongst lay public, and lack of readiness at the level of researchers, regulators and IEC members.

Despite the apprehensions, most of the IEC members agreed that such studies should be conducted in India as they offer benefits over animal studies and help to fast-track vaccine development especially in a pandemic situation. This is in line with a recent public consultation on SARS-CoV2 conducted in the United Kingdom where the participants believed that a controlled infection is better than infection with a wild virus ^31^. However, the risks in terms of pathogenicity of the controlled viral strain, severity of the disease, public health priority of the disease and availability of rescue treatment will have to be closely evaluated before agreeing to any specific study proposal on CHIM study ^32^. Further, as pointed out by Gordon *et al*, CHIM studies in LMICs such as India need to maintain standards equivalent to those prevalent internationally, and the risk to study participants should not be more than those elsewhere in the world.

The risk mitigation measures suggested by the members include strengthening of the regulatory, monitoring and ethical frameworks, and engagement of stakeholders to increase the credibility and understanding of CHIM studies. Some of the salient points included stringent selection criteria, a two-way consent process (including counselling wherever needed) to ensure comprehension (with adequate documentation and recording), reduction of risk of infection to family and society at large by ensuring proper infection control and appropriate biomedical waste disposal, strict post-study follow-up of volunteers, revised reimbursement and compensation guidelines and comprehensive insurance coverage for volunteers research team and IEC members. Other measures for risk reduction include capacity building and training of research team and IECs, which will approve such studies including experience sharing from CHIM-experienced research teams and IECs abroad.

Our study confirms these gaps which have been identified in previous published literature on CHIM studies in India ^1, 33^. Our results also emphasize the previous recommendations that CHIM studies should not be started before adequate community engagement has been done and public trust has been built ^34^.

Findings of our study resonate with the lessons learnt during implementation of CHIM studies in other LMICs ^35^ and the ethical framework suggested by Gordon *et al.* in Malawi ^6^.

Building the capacities of Ethics Committees, researchers, regulatory bodies through workshops, interactive and engaging consultations to manage the complexities of CHIM research is an ongoing process. Countries such as Kenya, Malawi, Vietnam and Zambia show us how such capacity is built ^36, 37^.

This is the first systematic multi-centric study amongst IEC members in the Indian setting to understand their perceptions about CHIM studies and their possible conduct in India. The study provides useful insights into the apprehensions about as well as the acceptance of CHIM studies by these key stakeholders. Theirs is a conditional acceptance, provided the required regulatory and ethical framework were in place in India. It was heartening to find in our qualitative exploration that IEC members were receptive, and engaged openly with the topic of CHIM studies, despite it being a new field to most of them and one with several layers of complexity and challenges. Within the duration of the FGDs, the tone of the discussions changed from trepidation to conditional acceptance as new thoughts and suggestions emerged on how to limit risks. There was a dynamic weighing of pros and cons with benefits and critical conditions needed for CHIMs in India evolving organically. This is reflective of the consideration of benefit and evaluating risk, when determining the justification of research protocols by IECs ^38^. However, the study included IEC members from only select research institutions (both government and private) in India, which are well-known for expertise in conducting clinical research and having well-established, accredited IECs. These opinions may not therefore be representative of other IECs in other research institutions or teaching hospitals in the country. Secondly, all the interviews were conducted during the COVID-19 pandemic and this might have influenced the perceptions compared to if the study had been done at another time. This was the first exploratory study amongst IEC members and did not focus on CHIM study for any particular disease. Future studies may look into risk and benefits of specific CHIM models of diseases of public health importance from the point of view of general public, IEC members or researchers.

Many of the concerns identified during this study have been addressed in the recently published WHO guidance on ethical conduct of CHIM studies, and that document will hopefully act as a roadmap for providing country specific guidance or regulations ^16^. A need for the harmonization of guidelines for CHIM studies across the world has been expressed ^39^. However, such international initiatives do not take away from the imperative of having our own guidelines in India, specific to our societal conditions and medical research milieu. Our study findings from a key stakeholder perspective of members of Indian IECs could pave the way in preparing the regulatory and ethical guidelines for implementation of CHIM studies in India.

Finally, much training, infrastructure development and strengthening of processes need to be in place before the introduction of CHIM studies in India can be considered safe and ethical. Public engagement and clear regulatory guidelines based on basic ethical principles can aid in creating a sustainable model for CHIM research. The establishment of required infrastructure with mandatory clinical units and residential facilities for the participants would be a key step, since the participants need to spend an average of 10–15 days in the facility. Rigorous safety standards are needed for CHIM studies especially owing to the infectious nature of the pathogens. These include mechanisms to ensure that participants do not come into direct contact with others and there is appropriate bio-medical waste disposal in the facility. It is suggested that both public and private institutions can be involved in conducting CHIM studies, and that such studies be funded by government bodies, to prevent allurement of institutions and investigations by commercial firms.

## Conclusion

In conclusion, members of IECs believe that it is important to conduct CHIM studies in India; however, before this is done, we would need to develop a specific regulatory and ethical framework for such studies, train research staff and IEC members, and put in place specialized research infrastructure, besides ensuring adequate community sensitization. Clinical infrastructure in terms of equipment, personllem and facilities are a non-negotiable basic as medical safety of the research participants is non-negotiabe. Our findings directs us towards a realization that preparedness for something as new and challenging as CHIM studies in a context such as India, requires readiness of multiple stakeholders, not sequentially but in parallel with each one of the constituent stakeholders having an influence over the other. Is it regulations that need to come first or public advocacy? Clinical and research infrastructure or training of all personnel involved? These are chicken and egg situations with no answer to be even attempted. This study has been critical in influencing a bottom up understanding of readiness of Ethics Committees not in isolation but with a number of other conditions in play. The conditions of the road, the skills of the driver, the availability of the Police, the functioning of the traffic lights and the agency and civic sense of the general public are all essential for a smooth ride!.

## Data Availability

The authors confirm that, for ethical and security reasons, they are unable to make interview transcripts and internal administrative documents publicly available. As outlined in the consent forms, interview respondents were informed that the data would be shared without revealing individual identities and with other researchers after approval by relevant local and national review committees. Requests for these data can be sent to the corresponding author [ abhishekanthropu@gmail.com]. Access to these restricted data will be granted where deidentification can be adequately achieved to protect the privacy and confidentiality of the respondents and any mentioned individuals and institutions, and where the proposed use is seen as relevant to the nature of the data. Figshare: FGD and IDI Guide. https://doi.org/10.6084/m9.figshare.20077346.v1 ^23^. This project contains the following extended data: ANNEXURE-I FGD GUIDE.pdf (Topic guide for FGD (focus group discussion)). ANNEXURE-II IDI GUIDE.pdf (Topic guide for IDI (in-depth interview)). Data are available under the terms of the Creative Commons Attribution 4.0 International license (CC-BY 4.0). Figshare: Presentation_CHIM.pptx. https://doi.org/10.6084/m9.figshare.20077751 ^24^ This project contains the following extended data: Presentation_CHIM.pptx (CHIM information that was presented to the IEC members before the FGD and IDI). Data are available under the terms of the Creative Commons Attribution 4.0 International license (CC-BY 4.0). Figshare: SRQR checklist for “Perceptions about controlled human infection model (CHIM) studies among members of ethics committees of Indian medical institutions: A qualitative exploration”. https://doi.org/10.6084/m9.figshare.20131826.v1 ^40^ Data are available under the terms of the Creative Commons Attribution 4.0 International license (CC-BY 4.0).
