# Supplementary material for: Perceptions about controlled human infection model (CHIM) studies among members of ethics committees of Indian medical institutions: A qualitative exploration
Source: Wellcome Open Res. 2023 Feb 28;7:209. Originally published 2022 Aug 12. [Version 2] doi: 10.12688/wellcomeopenres.17968.2 (PMC10031138; doi:10.12688/wellcomeopenres.17968.2)
Supplement: Peer review history

# Peer review: Reviewer response for version 2

**DOI:** 10.21956/wellcomeopenres.20878.r55318

**Published:** 2023-03-21

**Authors:** Manisha Gore

**Affiliations:** 1 Symbiosis Institute of Health Sciences, Symbiosis International (Deemed) University, Pune, Maharashtra, India

The author has addressed all the comments. However, I am of the opinion that there is still scope for further analysis and a comprehensive conceptual framework can certainly emerge which could guide the development of the theory. Yet in saying so, it would have its own limitations considering that this is a new study.

Is the work clearly and accurately presented and does it cite the current literature?

Partly

If applicable, is the statistical analysis and its interpretation appropriate?

Not applicable

Are all the source data underlying the results available to ensure full reproducibility?

No

Is the study design appropriate and is the work technically sound?

Partly

Are the conclusions drawn adequately supported by the results?

No

Are sufficient details of methods and analysis provided to allow replication by others?

Partly

Reviewer Expertise:

Public Health, Medical Anthropology

I confirm that I have read this submission and believe that I have an appropriate level of expertise to confirm that it is of an acceptable scientific standard.

# Peer review: Reviewer response for version 1

**DOI:** 10.21956/wellcomeopenres.19914.r52913

**Published:** 2022-11-08

**Authors:** Jeff D'Souza

**Affiliations:** 1 Institute for Better Health, Trillium Health Partners, Mississauga, ON, Canada

This manuscript provides timely and important insights into key stakeholders’ perceptions, views, and readiness to review research involving controlled human infection model (CHIM) studies in India. I strongly recommend its approval. This study makes a significant contribution to the academic literature and will be of great use to policymakers and others hoping to introduce CHIM studies in new settings.

 The following points are suggestions intended to strengthen the quality and contents of the submission. They should be viewed as “food for thought” but not as requirements for publication.  

1. The principle of ‘non-maleficence’ is a fundamental principle of medical ethics and involves a duty that a physician has toward their patients. When this principle is extended and adopted to a research ethics context, especially for non-therapeutic research, it is unclear – and a matter of vigorous academic debate – whether asking participants to partake in a study involving minimal risk (or risk slightly above minimal risk) violates the principle of non-maleficence. Here, I’d encourage the authors to rethink stating without qualification that CHIM studies violate the principle of non-maleficence. If this is the view of the authors, perhaps further substantiation could be provided.
2. It would be good to know whether participants’ views, perceptions, and readiness to review CHIM studies depends on the level of risk or the kind of CHIM study. Some of the feedback shared clearly have to do with CHIM studies involving more than a slight increase above minimal risk, where rescue treatments are needed, and where there is a risk of long-term harm or disease. However, views, perceptions and readiness may change if the CHIM studies under consideration were deemed to be slightly above minimal risk. If such information could be provided, that would be helpful. 
3. Some discussion on what it is about CHIM studies that warrants special ethics and regulatory attention would be helpful to situate the paper, and guide readers in their understanding of how, for example, a CHIM study is different than a first-in-human phase 1 drug trial.
4. I would encourage the authors to build upon this important work.  One possible route they may take is to draw a distinction among the various themes and challenges raised and determine whether such themes and challenges are (i) ethics/research ethics issues or (ii) practical issues, and then come up with remedies and solutions to adequately address them. Given the potential social and societal benefit of conducting CHIM studies in India this seems to be one potential next step.  Another possible route the authors might take is to conduct a similar study where they engage research ethics committee members' perceptions, views and readiness to review specific CHIM studies for particular diseases and pathogens that have a high burden of disease in India, and where CHIM studies may provide significant scientific and social value. This kind of more specific feedback may be particularly helpful with the introduction of CHIM studies that investigate particular diseases/pathogens in India.   

To reiterate, the four comments above are simply suggestions. Overall, this is an excellent manuscript that makes a significant contribution to the academic literature. While community engagement in India on the introduction of CHIM studies has been going on for some time, this is the first study of its kind to systematically engage research ethics committee members directly – i.e., the individuals who have special responsibilities to safeguard the rights, safety, and welfare of research participants.

Is the work clearly and accurately presented and does it cite the current literature?

Yes

If applicable, is the statistical analysis and its interpretation appropriate?

Not applicable

Are all the source data underlying the results available to ensure full reproducibility?

No source data required

Is the study design appropriate and is the work technically sound?

Yes

Are the conclusions drawn adequately supported by the results?

Yes

Are sufficient details of methods and analysis provided to allow replication by others?

Yes

Reviewer Expertise:

Global Health Ethics; Research Ethics; Clinical Ethics

I confirm that I have read this submission and believe that I have an appropriate level of expertise to confirm that it is of an acceptable scientific standard.

# Response

**Published:** 2023-01-04

**Authors:** Abhishek Sharma

**Affiliations:** HelpAge India, India

Thank you so much for these very encouraging comments The following are the point by point responses to the comments.  

 1. The following points are suggestions intended to strengthen the quality and contents of the submission. They should be viewed as “food for thought” but not as requirements for publication. The principle of ‘non-maleficence’ is a fundamental principle of medical ethics and involves a duty that a physician has toward their patients. When this principle is extended and adopted to a research ethics context, especially for non-therapeutic research, it is unclear – and a matter of vigorous academic debate – whether asking participants to partake in a study involving minimal risk (or risk slightly above minimal risk) violates the principle of non-maleficence. Here, I’d encourage the authors to rethink stating without qualification that CHIM studies violate the principle of non-maleficence. If this is the view of the authors, perhaps further substantiation could be provided.  

**Response-** Thank you for this comment. The whole paper is about ethically contentious issues and perceptions of maleficence. We feel it is important to state the contentions upfront. We have now reframed the line as follows: A disease-causing pathogen is for the purpose of study, intentionally inoculated into healthy, consenting adult volunteers, with no individual benefit to them1, a possible violation of the principle of non-maleficence. The degree of it could vary depending on the pathogenicity of the virulent introduced, the dosage and the preparedness to manage its onslaught on the body and the effectiveness of the treatment available. This issue of non-maleficence is heightened further when there is no treatment available for the infection.  

 2. It would be good to know whether participants’ views, perceptions, and readiness to review CHIM studies depends on the level of risk or the kind of CHIM study. Some of the feedback shared clearly have to do with CHIM studies involving more than a slight increase above minimal risk, where rescue treatments are needed, and where there is a risk of long-term harm or disease. However, views, perceptions and readiness may change if the CHIM studies under consideration were deemed to be slightly above minimal risk. If such information could be provided, that would be helpful.  

**Response-** You are absolutely right in asking this as to how the participant’s views changed based on the level of the risk. This makes for an excellent research question for a follow-up study with larger sample sizes, and multiple vignettes with scales developed. Since this was the first exploration in India, we were unable to assess this in this study. We have to note that this study was looking at IEC members’ perceptions of hypothetical CHIM scenarios and was being cautious and looking at worst case situations to protect research subjects. Future studies could look at specific comparisons of ‘high risk’ and ‘low risk’ CHIM studies and the differences in responses.            

 3. Some discussion on what it is about CHIM studies that warrants special ethics and regulatory attention would be helpful to situate the paper, and guide readers in their understanding of how, for example, a CHIM study is different than a first-in-human phase 1 drug trial.  

**Response-** Thank you. We have added the following sentence in the discussion:  CHIM studies are different from first in human studies as these studies don’t just involve exposure to new drug but include establishment of a disease model in healthy humans using a standardized pathogen and then evaluating the effects of drugs and vaccines within the established disease model. This is in the context where people outside the study have already suffered from the pathogen as against a new drug which has never been used before.  

 4. I would encourage the authors to build upon this important work. One possible route they may take is to draw a distinction among the various themes and challenges raised and determine whether such themes and challenges are (i) ethics/research ethics issues or (ii) practical issues, and then come up with remedies and solutions to adequately address them. Given the potential social and societal benefit of conducting CHIM studies in India this seems to be one potential next step.  Another possible route the authors might take is to conduct a similar study where they engage research ethics committee members' perceptions, views and readiness to review specific CHIM studies for particular diseases and pathogens that have a high burden of disease in India, and where CHIM studies may provide significant scientific and social value. This kind of more specific feedback may be particularly helpful with the introduction of CHIM studies that investigate particular diseases/pathogens in India.  

**Response**- Thank you for this comment and your positive feedback.  We will definitely take up these suggestions for the next phase of studies. We have added the following sentence in the discussion section: This was the first exploratory study amongst IEC members and did not focus on CHIM study for any particular disease. Future studies may look into risk and benefits of specific CHIM models of diseases of public health importance from the point of view of general public, IEC members or researchers.

# Peer review: Reviewer response for version 1

**DOI:** 10.21956/wellcomeopenres.19914.r52574

**Published:** 2022-10-20

**Authors:** Manisha Gore

**Affiliations:** 1 Symbiosis Institute of Health Sciences, Symbiosis International (Deemed) University, Pune, Maharashtra, India

Main comments:

1. Introduction requires strengthening of the rationale for study.
2. Overlapping of methods (FGD-IDI) needs to be justified.
3. Major flaw in the analysis of the qualitative data.
4. Lack of clarity in the process of transfer of codes to themes.
5. Grounded theory approach: absent of the theory/ theoretical framework.
6. For heterogeneous participants, context differs accordingly perceptions will differ.
7. I recommend separate analysis (site-wise) and then building on it. Should adopt triangulation. 
8. For such type of research expect a detailed conclusion with recommendations.

Additional comments:

- The study mentions that FGDs were conducted to understand the deliberative process of decision-making in IEC meetings and IDI to understand perceptions about CHIM- analysis could have been done independently for both sources.
- Heterogeneity among the participants as clinicians (CN), biomedical scientists (BM), social scientists (SS), legal experts (LE), and lay persons (LP). Context-specific analysis with respect to the expertise of the IEC members could have been done rather than a common analysis of data of IDI considering that perceptions are subjective and would vary with the educational background and expertise.  
- Themes are not properly constructed; they are broad and moreover are like headings of the paragraphs.
- There is no category development, and there is an absence of a coding framework (should have a table explaining the process from code-categories-themes).
- Triangulation of data from different sources/participants is not attempted.
- A substantive theory is produced in order to present the findings of a Grounded Theory - as a collection of concepts connected to one another in an interconnected whole. The absence of a theoretical framework/theory is the biggest lacuna of the study.

Is the work clearly and accurately presented and does it cite the current literature?

Partly

If applicable, is the statistical analysis and its interpretation appropriate?

Not applicable

Are all the source data underlying the results available to ensure full reproducibility?

No

Is the study design appropriate and is the work technically sound?

Partly

Are the conclusions drawn adequately supported by the results?

No

Are sufficient details of methods and analysis provided to allow replication by others?

Partly

Reviewer Expertise:

Public Health, Medical Anthropology

I confirm that I have read this submission and believe that I have an appropriate level of expertise to state that I do not consider it to be of an acceptable scientific standard, for reasons outlined above.

# Response

**Published:** 2023-01-04

**Authors:** Abhishek Sharma

**Affiliations:** HelpAge India, India

The following are the point by point responses to the comments.  

 1. Introduction requires strengthening of the rationale for study.    

 The Introduction states the ethical challenges in CHIM studies, namely the possible intentional harm of introducing a pathogen into a healthy consenting study volunteer, the vulnerability of study volunteers especially considering the socio-economic situation of India and hence the important role played by the Institutional Ethics Committee, the  key functionary in the protection of human volunteers as mandated by all ethical regulatory bodies and also the Indian Council of Medical Research. To reiterate the rationale the following line has been added to the end of the Introduction:  The present study has been carried out amongst consenting members of IECs from multiple health care research institutes in India with experience in conducting clinical trials to elicit their views on the ethics of conducting CHIM studies in India.   Following text has been added to the introduction section: A disease-causing pathogen is for the purpose of study, intentionally inoculated into healthy, consenting adult volunteers, with no individual benefit to them1, a possible violation of the principle of non-maleficence. The degree of it could vary depending on the pathogenicity of the virulent introduced, the dosage and the preparedness to manage its onslaught on the body and the effectiveness of the treatment available. This issue of non-maleficence is heightened further when there is no treatment available for the infection.    

 2. Overlapping of methods (FGD-IDI) needs to be justified.

 Thank you for this comments. FGDs and IDI, played a complementing role. While FGDs provided the dynamic process of discussion and deliberation similar to the real world functioning of IECs in reviewing research proposals, the IDIs helped to provide a more in-depth perspective of individual members. Provision of allowing IDI also allowed views of a member who could not make it on the day of the FGD to express his/her views. One method helped the other and hence the need to have these overlapping methods. We have added the above text to the methods.

# Response

**Published:** 2023-01-04

**Authors:** Abhishek Sharma

**Affiliations:** HelpAge India, India

3. Major flaw in the analysis of the qualitative data.  

**Response:** We are not very clear on this query and details would be desirable but would like to add the following: On the whole, the methods, as in done in qualitative research were tailored to best suit our research question. This is in contrast with the straight jacket methods in quantitative methods.  

 4. Lack of clarity in the process of transfer of codes to themes.    

**Response:** This was an iterative process as demanded by qualitative research and took place at multiple levels and over a period of time within sites and between sites and has been presented in the Figure 2. Generating themes.  

 5. Grounded theory approach: absent of the theory/ theoretical framework.  

**Response:** We have used the principles of grounded theory to guide the process of iterative analysis of the data. As there was no theoretical framework for this research question, we had to start by a thematic analysis and building it from the data. The work of Strauss A, Corbin JM: Basics of qualitative research: Grounded theory procedures and techniques. Thousand Oaks, CA, US: Sage Publications, Inc; 1990; 270 has been used to guide the GT approach followed and has been explained with this line This exploration is referred to as 'grounded' since they are based on the participants' own explanations or interpretations. An inductive approach ensures that the researcher begins with an almost blank slate and considers the opinions of the participants rather than imposing one’s own. The following line has been added: This was important as CHIM studies are new and de novo rather than a priori ethical responses to them were being sought.  

 6. For heterogeneous participants, context differs accordingly perceptions will differ. **Response:** Yes, traditionally FGDs require participants to be homogenous, that means all clinicians with similar experience or all legal experts with similar experience, or all social workers with similar experience, etc. This group interview was specific to the research question we aimed to answer, “ what are the ethical issues which come to the mind of members of ethics committees ?. Here, in this setting by definition each member will look at it from difference lenses based on their disciplinary backgrounds, and that’s what we are looking at exploring, and documenting. While participants appear heterogeneous in terms of their individual backgrounds and domains of expertise, they are homogenous in terms of the purpose of coming together and being selected as IEC members ie. the protection of human volunteers participating in biomedical research and the upholding of ethical standards in human participant research.  

 7. I recommend separate analysis (site-wise) and then building on it. Should adopt triangulation.  

**Response:** Thank you for the suggestions. We deliberated on this same matter and concluded that we did not want to draw inferences state wise or make comparisons across institutions but rather look at emerging viewpoints across all sites across India. All ethics committees adopt the same principles of thinking and functioning as all international ethics regulatory bodies and principles  which are also aligned to the  rules and regulations of the ICMR National Ethical Guidelines for Biomedical and Health Research involving human participants. We were keen to bring in views from different disciplinary backgrounds such as basic scientists, lawyers, clinicians etc. Also another consideration was confidentiality. Any further details – site specific would break the code of confidentiality, which we had signed on when seeking consent from the members of the ethics committees at each site.  We did not want a any site specific  disclosure  or  individual identities. We did not want to jeopardise the ethical premise of the confidentiality of data and processes.  

 8. For such type of research expect a detailed conclusion with recommendations. **Response:** This study was not powered to be normative or prescriptive but to be descriptive and provide direction for future work – research, training, development stringent code of conducts to suit CHIM studies , or fine tuning of guidelines and public engagement. This has been spelt out in Figure 3. Proposed risk mitigation measures for CHIM studies. A line summarising the above figure has been brought into the paragraph on Conclusions. In conclusion, our grounded theory approach to our findings directs us towards a realisation that preparedness for something as new and challenging as CHIM studies in a context such as India, requires readiness of multiple stakeholders, not sequentially but in parallel with each one of the constituent stakeholders having an influence over the other. Is it regulations that need to come first or public advocacy? Clinical and research infrastructure or training of all personnel involved? This study has been critical in influencing a bottom up understanding of readiness of Ethics Committees not in isolation but  with a number of other conditions in play. For example introducing formula racing – one has to also consider the conditions of the road, the skills of the driver, the availability of the Police, the functioning of the traffic lights and the agency and civic sense of the general public are all essential for a smooth ride!

# Response

**Published:** 2023-01-04

**Authors:** Abhishek Sharma

**Affiliations:** HelpAge India, India

**Response to Additional comments:**

 ● The study mentions that FGDs were conducted to understand the deliberative process of decision-making in IEC meetings and IDI to understand perceptions about CHIM- analysis could have been done independently for both sources.  

**Response: **This has been addressed earlier “ FGDs and IDI, played a complementing role. While FGDs provided the dynamic process of discussion and deliberation similar to the real world functioning of IECs in reviewing research proposals, the IDIs helped to provide a more in-depth perspective of individual members. Provision of allowing IDI also allowed views of a member who could not make it on the day of the FGD to express his/her views. One method helped the other and hence the need to have these overlapping methods.” As mentioned earlier, these were overlapping methods. The codes and themes were generated using iterative methods.

 ● Heterogeneity among the participants as clinicians (CN), biomedical scientists (BM), social scientists (SS), legal experts (LE), and lay persons (LP). Context-specific analysis with respect to the expertise of the IEC members could have been done rather than a common analysis of data of IDI considering that perceptions are subjective and would vary with the educational background and expertise. 

**Response:** As mentioned earlier,  while participants appear heterogeneous in terms of their individual backgrounds and domains of expertise, they are homogenous in terms of the purpose of coming together and being selected as IEC members … ie. the protection of human volunteers participating in biomedical research and the upholding of ethical standards in human participant research. We had to also be sensitive to maintaining the confidentiality of the sites and study participants. Ethics committee composition is pubic knowledge, so any more details would be breaking the code of confidentiality about who has said what from which committee.  

 ● Themes are not properly constructed; they are broad and moreover are like headings of the paragraphs.

**Response:** Being the first exploratory study from India, the themes were built from bottom up.

    

 ● There is no category development, and there is an absence of a coding framework (should have a table explaining the process from code-categories-themes).

**Response:** Subthemes used in the analysis are analogous to categories as they have emerged from the codes and feed into emerging themes. Please refer to figure 1 and 2 of the manuscript that explain the methods in details.  

 ● Triangulation of data from different sources/participants is not attempted.  

**Response: **We have used overlapping methods i.e. in-depth interviews and focused group discussion for this purpose. Also, data has been collected from different members of the IEC that play distinct roles in the decision making while conducting ethical review.      

 ● A substantive theory is produced in order to present the findings of a Grounded Theory - as a collection of concepts connected to one another in an interconnected whole. The absence of a theoretical framework/theory is the biggest lacuna of the study.  

**Response:** We have used the principles of grounded theory to guide the process of iterative analysis of the data. As there was no theoretical framework for this research question, we had to start  with a thematic analysis and building from the data.
